# A case of *Mycobacterium goodii* prosthetic valve endocarditis in a non-immunocompromised patient: use of 16S rDNA analysis for rapid diagnosis

**DOI:** 10.1186/1471-2334-12-301

**Published:** 2012-11-14

**Authors:** Göran Jönsson, Johan Rydberg, Erik Sturegård, Bertil Christensson

**Affiliations:** 1 Department of Clinical Sciences, Division of Infection Medicine, University Hospital of Skåne, Lund SE-221 85, Sweden; 2 Department of Laboratory Medicine, Division of Microbiology, Lund, SE-221 85, Sweden; 3 Department of Clinical Microbiology, Laboratory Medicine Skåne, Lund University, Malmö, SE-205 02, Sweden

**Keywords:** *Mycobacterium goodii*, NTM, Endocarditis, Septic emboli, Prosthetic valve, 16S rDNA analysis

## Abstract

**Background:**

*Mycobacterium goodii* is a rare cause of significant infection. *M*. *goodii* has mainly been associated with lymphadenitis, cellulitis, osteomyelitis, and wound infection.

**Case presentation:**

A case of a 76-year-old Caucasian female is presented. The patient developed a prosthetic valve endocarditis caused by *M*. *goodii*. She had also suffered from severe neurological symptoms related to a septic emboli that could be demonstrated as an ischemic lesion found on CT of the brain. Transesophageal echocardiography verified a large vegetation attached to the prosthetic valve. Commonly used blood culture bottles showed growth of the bacteria after 3 days.

**Conclusions:**

Although *M*. *goodii* is rarely involved in these kinds of severe infections, rapidly growing mycobacteria should be recognized during conventional bacterial investigations and identified by molecular tools such as analysis of 16S rDNA. Species identification of nontuberculous mycobacteria is demanding and is preferably done in collaboration with a mycobacterial laboratory. An early diagnosis provides the opportunity for adequate treatment. In the present case, prolonged antimicrobial treatment and surgery with replacement of the prosthetic valve was successful.

## Background

*Mycobacterium goodii* was identified in 1999 as a rapidly growing non tuberculous mycobacteria (NTM) species related to the *Mycobacterium smegmatis* group isolated from human wound infections [1]. *M*. *goodii* has previously been associated with sporadic cases of cellulitis, osteomyelitis, infected pacemaker sites, lipoid pneumonia [1,2], and bursitis [3]. *M*. *goodii* has also been related to minor hospital outbreaks in patients receiving surgical implants [4]. However, there has been no reported case of native or prosthetic valve endocarditis due to *M*. *goodii*. Since 1978, there are at least 9 cases reported in the literature with native and prosthetic valve endocarditis recognized to belong to the *M*. *fortuitum* group [5]. Some of these patients were probably more susceptible to this type of infection due to underlying medical conditions such as HIV, intravenous drug use and renal failure [5]. Here we present a case of prosthetic valve endocarditis caused by *M*. *goodii*.

## Case presentation

A 76-year-old Caucasian female was admitted to our hospital in September 2009 with a sudden onset of dyspnea that had emerged during dancing a foxtrot. She was previously remarkably healthy and lived a very active life with hobbies like dancing and gardening. On initial assessment at the Emergency Department, she had no history of chills, fever, night sweats, cough or chest pain. Physical examination revealed a severe respiratory distress requiring oxygen treatment. Ultrasound examination of the heart was consistent with a mitral valve insufficiency caused by a rupture of chordae tendineae. Coronary angiography was normal except for a significant proximal stenosis found in D2. Eleven days after admission (September 28) the patient received a biological mitral valve replacement (Edward Magna Nr 25, Edwards Lifesciences Corp, Irvine, CA, USA) and a coronary artery bypass of D2 was also performed. The postoperative course was uneventful, and the patient was discharged from the hospital in good health on the 5th of October.

On the 15th of January 2010, the patient was readmitted to the Department of Neurology. The patient had suffered from general fatigue for at least 2–3 weeks, and had experienced slurred speech, poor balance, and disorientation to time and places for at least 24 hours. Initial neurological examination showed difficulty in walking due to poor balance. She was afebrile. Computed tomography (CT) of the brain revealed an ischemic lesion of the right cerebellar hemisphere, and she was assumed to have suffered from episodes of transient ischemic attacks and stroke. No blood cultures were drawn, and the patient was discharged. Further investigation as an out-patient revealed normal results from carotid Doppler, electroencephalography, and Holter ambulatory electrocardiography.

The patient was readmitted to the Emergency Department on the 2nd of February 2010. She was again confused with slurred speech, complained about general weakness, and had now a temperature of 40.2°C. Her heart rate was 99 beats per minute without cardiac murmurs, the blood pressure was 100/45 mmHg, and further physical examination was normal. Laboratory investigations showed the following results: hemoglobin 101 g/L, leukocyte count 7.1 × 10^9^/L (ref. 4–10 × 10^9^/L), platelet count 155 × 10^9^/L (ref. 140–400 × 10^9^/L), and C-reactive protein (CRP) was 110 mg/L (ref. <3.0 mg/L). Serum sodium, potassium and creatinine were within normal ranges. After securing four sets of blood cultures, the patient was initially given empirical treatment for bacterial meningitis with intravenous ampicillin, cefotaxime, and betamethasone. However, she developed no stiffness of the neck and further analysis of cerebrospinal fluid showed no signs of meningitis. Also, chest radiography was normal, and CT of the brain did not reveal any new lesions including abscesses. Transesophageal echocardiography (TEE) was inconclusive with an unclear structure found floating in the left auricle. No vegetation or insufficiency was described. The patient rapidly improved, became afebrile and was discharged after two days of hospitalization. She was prescribed oral amoxicillin 500 mg t.i.d. for 7 days.

**Figure 1:**
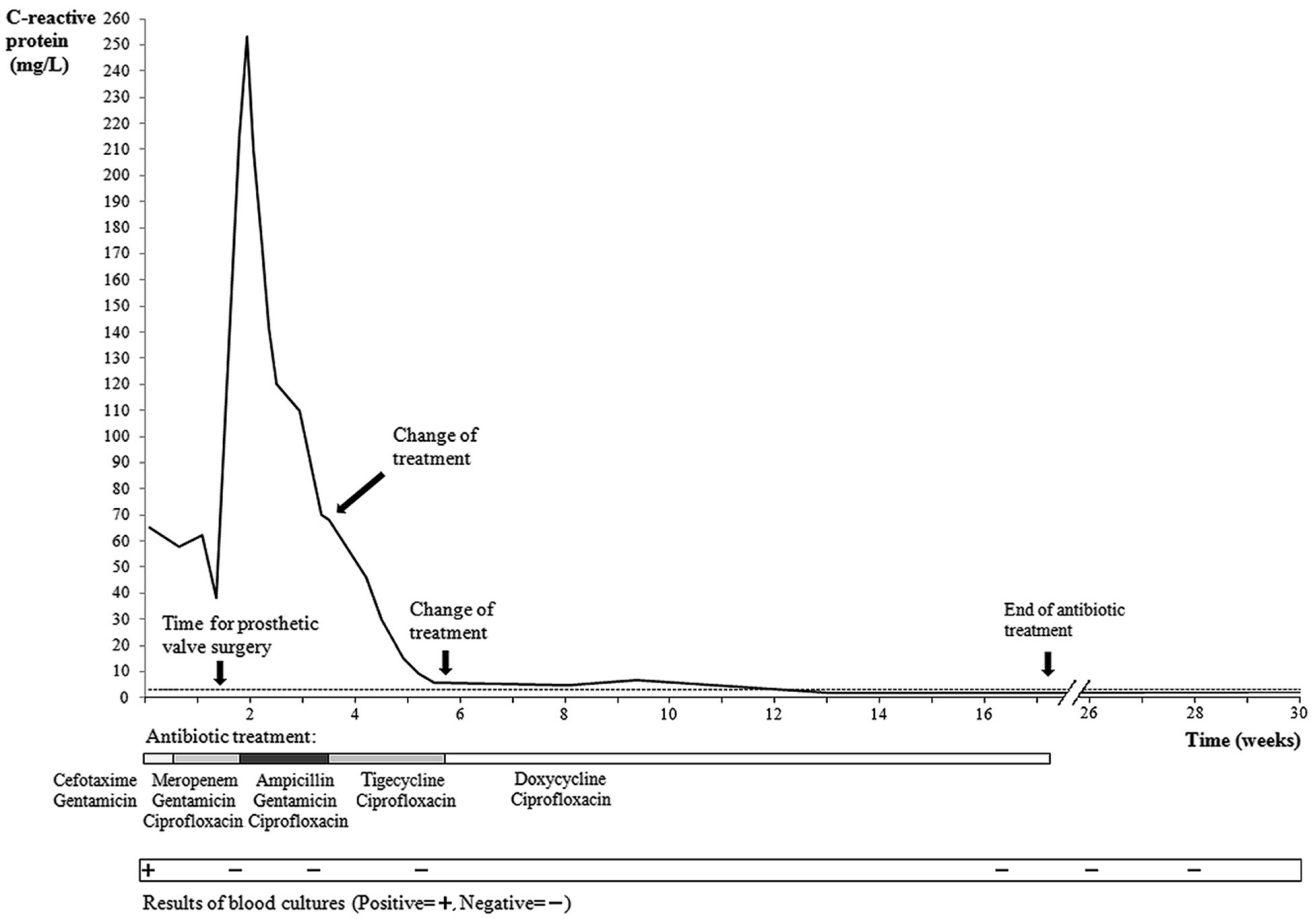
**Comparison between C-reactive protein (CRP) levels and treatments given over time.** The first antibiotic regime was cefotaxime and gentamicin administered for three days. After the prosthetic valve surgery, the patient received antibiotics for four months. Twelve weeks after surgery, CRP was found to be repeatedly at a normal level. Only the initially drawn blood cultures showed growth of *Mycobacterium goodii*. The broken line indicates CRP ≤ 3.0 g/L.

After five days (February 9) the patient returned with general fatigue, confusion and fever of 40°C, again with similar results of physical, neurological and laboratory examinations as on February 2nd. A new TEE demonstrated a large (13 mm), mobile vegetation attached to the prosthetic mitral valve. Treatment for endocarditis with cefotaxime and gentamicin was instituted. The blood cultures, drawn on the 2nd of February, were inoculated in aerobic and anaerobic blood culture bottles (bioMerieux, FAN-bottles, Marcy l’Etoile, France), and one aerobic bottle indicated growth after 3 days. A catalase positive Gram-positive rod with partial Gram-staining was identified on blood agar plates. It was initially considered to be a diphteroid rod with doubtful clinical significance, but when all four blood culture sets subsequently showed growth of multidrug-resistant Gram-positive rods, 16S rDNA PCR analysis was performed [6]. Sequence analysis covering ~800 bp of the 16S rRNA gene demonstrated a complete homology with *M*. *goodii* isolates from GenBank. Once it was clear that the isolate was a rapidly growing mycobacterial species, the strain was sent to the mycobacterial section at the clinical microbiology laboratory. Species identification of *M*. *goodii* using 16S rDNA sequencing was confirmed using Genotype® Mycobacterium CM and AS assays (HAIN Lifescience, Nehren, Germany). Antimicrobial susceptibility testing (AST) was performed in two stages, first at the conventional bacteriology section, followed by additional testing at the mycobacteriology section. All AST were performed with use of Etest® (bioMérieux, Marcy l’Etoile, France) on Mueller Hinton agar. At this stage, the treatment was changed to meropenem combined with gentamicin and ciprofloxacin (Figure 1) based on the antibiotic susceptibility pattern (Table 1). The fever resolved and CRP declined from 65 mg/L to 38 mg/L.

**Table 1:** **The antibiotic resistance pattern of the *Mycobacterium goodii *isolated from blood cultures**

**Antibiotic substance**	**MIC (mg/L)**
Amikacin	0.5
Ampicillin	0.5
Benzylpenicillin	>256
Cefotaxime	>256
Ciprofloxacin	0.125
Clarithromycin	128
Clindamycin	64
Ertapenem	>32
Erythromycin	256
Fusidic acid	256
Gentamicin	0.5
Imipenem/cilastatin	4
Linezolid	4
Meropenem	2
Moxifloxacin	0.032
Rifampicin	64
Tetracycline	0.25
Tigecycline	0.016
Tobramycin	1
Trimethoprim/sulfamethoxazole	0.125
Vancomycin	16

The patient went through mitral valve replacement surgery on the 19th of February, again with a similar biological prosthetic valve (Edward Magna Nr 25, Edwards Lifesciences Corp, Irvine, CA, USA). The rationales for this decision were the finding of a prosthetic valve endocarditis due to a highly resistant microorganism and a large vegetation with a risk of new episodes of embolization. At surgery a vegetation was found and there were signs of infection surrounding the prosthetic valve ring. Cultures from the vegetation showed no growth, but 16S rDNA gene sequencing was consistent with the findings of *M*. *goodii*. After surgery there was a dramatic improvement of the patient’s general condition. Meropenem was replaced by ampicillin due to lower MIC-value (Table 1), but during the third week of treatment CRP was repeatedly found to be about 70 mg/L (Figure 1). The antibiotic treatment was changed in accord with the susceptibility pattern that favoured the combination of tigecycline and ciprofloxacin (Table 1). The following two weeks CRP dropped to 6.1 mg/L. The patient was discharged on March 23 in good health and continued with the combination of oral doxycycline and ciprofloxacin for an additional eleven weeks. She was followed clinically and with laboratory analyses for 30 weeks. CRP remained at a normal level and repeatedly drawn blood cultures showed no bacterial growth (Figure 1).

## Conclusions

Infective endocarditis caused by *M*. *goodii* has to our knowledge not previously been described. *M*. *goodii* is a rapidly growing NTM, and visible colonies are formed on Loewenstein-Jensen culture medium within 7 days of incubation [7]. Microbiological diagnosis is obtained by microscopy for acid-fast bacilli on secretions or biopsy, and by culture. Species identification of NTM is demanding and often a combination of biochemical and/or molecular methods needs to be applied. In many cases 16S rDNA-sequencing is enough for species identification but a number of NTMs have similar gene sequences enhancing the importance of several different approaches to identify NTMs at a species level [8].

NTMs are found in the natural environment throughout the world and include more than 135 different species. Rapidly growing NTMs have been isolated from potable and natural water, and in soil. They can also be isolated from contaminated tap water. There are evidence suggesting that nosocomial transmission have occurred for *M*. *fortuitum*, *M*. *chelonae*, *M*. *abscessus*, and *M*. *goodii*[4,9], which resulted in conditions ranging from harmless colonization to invasive infection. One explanation to this problem might be that many of the nontuberculous mycobacteria are resistant to disinfectants [9], and an effective disinfectant or antiseptic against *M*. *goodii* has not been reported.

NTM infections in surgical patients have been reported in a wide variety of settings. The use of colonized aqueous solutions and inadequate sterilization or disinfection of surgical equipment are often factors involved in these infections. *M*. *fortuitum* and *M*. *chelonae* have caused multiple outbreaks of sternal wound infection and endocarditis after cardiac surgery. One outbreak was traced to contaminated tap water that had been used to make cardioplegia solution. The highest mortality rates have been reported in patients with prosthetic valve replacements [10-13]. The implantation of porcine heart valves colonized with *M*. *chelonae* during manufacture resulted in pericarditis and endocarditis [14,15]. *M*. *smegmatis*, *M*. *chelonae*, *M*. *gordonae*, and *M*. *fortuitum* have caused isolated cases of prosthetic valve endocarditis in the immediate postoperative period. *M*. *chelonae*, *M*. *fortuitum*, and *M*. *smegmatis* have been retrieved in isolated cases of postoperative sternal wound infections and mediastinitis [13,16,17]. *M*. *chelonae* has been isolated from vein graft harvest site infections after cardiac bypass surgery [18].

Our patient had received a prosthetic mitral valve four months before the infection gave any obvious symptoms such as fever. It seems likely that our patient had a prolonged, indolent infection that was acquired during surgery, as indicated by reports of porcine aortic valves contaminated by other NTMs [14,15]. During the observed period (January 2010 to October 2011), there was no other patient found with a *M*. *goodii* infection at our hospital, suggesting no obvious environmental source of contamination. However, no further investigation regarding an environmental source was performed, and thus it can not be determined whether the valve prosthesis was contaminated before or during surgery. In retrospect, our patient had never really regained her strength after the first operation and had suffered from general fatigue. Also, we believe that her neurological symptoms already on January 15th, including the ischemic lesion found on CT of the brain, represent embolic lesions from the prosthetic endocarditis. The very late onset of clear signs of infection in our patient is different from what has been previously described in patients with *M*. *goodii* associated surgical implant infections (3 days-4 weeks) [4].

Antimicrobial susceptibility data for *M*. *goodii* are sparse. Brown et al. [1] showed that all *M*. *goodii* isolates were susceptible to amikacin, doxycycline, ciprofloxacin, and sulfamethoxazole, and were variably susceptible to cefoxitin and clarithromycin. These bacteria are usually also susceptible to ethambutol and exhibit good in vitro susceptibility to gatifloxacin and ciprofloxacin [2]. Commonly used antimicrobials include trimethoprim/sulfamethoxazole, doxycycline, and ciprofloxacin. Intravenous amikacin and imipenem have been used for severe cases [8]. However, there are few data on the clinical validation of using susceptibility breakpoints of tested drugs. Notably, tigecycline has to our knowledge not been previously used in the treatment of *M*. *goodii* disease, and in the present case we believe that tigecycline treatment contributed to the successful outcome. In prosthetic endocarditis, surgical excision of the valve is probably necessary besides treatment with a combination of antibiotics for about 4 months [8]. The in vitro susceptibility pattern observed for *M*. *goodii* in this investigation is consistent with previously reported findings. Notably, there are specific culturing techniques for the recovery of mycobacterial blood stream infections which are used when mycobacterial infection is highly suspected, but this case highlights that commonly used blood culture bottles such as bioMerieux, FAN-bottles may be sufficient to detect rapidly growing NTM.

In summary, *M*. *goodii* is a rapidly growing mycobacterium increasingly recognized as an important human pathogen. It is most commonly implicated in surgical site and posttraumatic infections, but is also associated with respiratory infections in patients with lipoid pneumonia. Identification of the organism is best accomplished by molecular techniques and 16S rDNA-sequencing is an example of a useful analysis in suspected NTM disease. Our patient history highlights the importance of rapidly performed testing for mycobacteria in prosthesis-related infections.

## Consent

Written informed consent was obtained from the patient for publication of this case and any accompanying imaged. A copy of the written consent is available by Series Editor of this journal.

## Abbreviations

CRP: C-reactive protein; CT: Computed tomography; PCR: Polymerase chain reaction; NTM: Non tuberculous mycobacteria; TEE: Transesophageal echocardiography.

## Competing interests

Prof. Bertil Christensson is a member of Pfizer Advisory Board for Bacterial Infections. Johan Rydberg, Erik Sturegård and Göran Jönsson declare no competing interests.

## Authors’ contribution

All authors of this case report made substantial contributions to conception and design. GJ and BC drafted the manuscript. JR and ES were involved in the microbiological investigations. All authors revised the manuscript critically, read, and approved the final version.

## Pre-publication history

The pre-publication history for this paper can be accessed here:

http://www.biomedcentral.com/1471-2334/12/301/prepub
